# Prognostic Significance of Actinin‐4 Protein Expression and Gene Amplification in Endometrial Carcinoma

**DOI:** 10.1111/jog.70132

**Published:** 2025-11-11

**Authors:** Li Xiang, Yutaka Naito, Masafumi Toyoshima, Mika Terasaki, Akihito Yamamoto, Akira Shimizu, Shunji Suzuki, Kazufumi Honda

**Affiliations:** ^1^ Department of Obstetrics and Gynecology Nippon Medical School Tokyo Japan; ^2^ Department of Molecular Prevention, Institute for Advanced Medical Science Nippon Medical School Tokyo Japan; ^3^ Department of Analytic Human Pathology Nippon Medical School Tokyo Japan; ^4^ Department of Molecular Prevention, Graduate School of Medicine Nippon Medical School Tokyo Japan

**Keywords:** actinin‐4, *ACTN4*, endometrial carcinoma, fluorescence in situ hybridization, immunohistochemical staining

## Abstract

**Objective:**

This study aimed to investigate the clinical significance of actinin‐4 in endometrial carcinoma. Actinin‐4, an actin‐binding protein involved in cytoskeletal dynamics, has been implicated in the progression of various cancers; however, its precise role in endometrial carcinoma is not fully understood. This research sought to evaluate actinin‐4 protein expression and gene amplification and correlate these findings with clinicopathological parameters and patient survival to determine its prognostic value.

**Methods:**

A retrospective analysis was conducted on endometrial carcinoma patients who underwent surgical resection. Actinin‐4 protein expression was assessed using immunohistochemical staining (IHC), and *ACTN4* gene amplification was evaluated by fluorescence in situ hybridization (FISH). The intensity of actinin‐4 staining was graded, and gene amplification of *ACTN4* was defined using the *ACTN4*/*CEP19* ratio. Statistical analysis, including Kaplan–Meier survival analysis and Cox proportional hazards modeling, was performed to correlate actinin‐4 expression with clinicopathological features and survival outcomes.

**Results:**

Overexpression of actinin‐4 protein by IHC was significantly associated with advanced clinical stage and histological subtypes. While no significant difference was observed in overall survival (OS), patients with high actinin‐4 IHC demonstrated significantly poorer progression‐free survival (PFS). *ACTN4* gene amplification by FISH was significantly associated with poorer prognosis for both OS and PFS compared to the group without amplification.

**Conclusion:**

This study suggests that actinin‐4 plays a role in the progression of endometrial carcinoma, particularly influencing tumor aggressiveness and progression‐free survival.

## 1 Introduction

Endometrial carcinoma, originating from the uterine lining, represents the most common gynecological malignancy in developed countries, with its incidence demonstrating a concerning upward trend [1]. While advancements in early detection and treatment strategies have contributed to improved overall prognosis, a significant subset of patients continues to face the risks of recurrence and metastasis [2]. This highlights a critical need for more precise prognostic markers to guide individualized treatment paradigms and optimize patient outcomes [3].

Current clinicopathological staging systems, despite their widespread use, have inherent limitations in fully capturing the complex heterogeneity of endometrial carcinoma [4]. This can lead to suboptimal treatment decisions, potentially resulting in both under‐ and over‐treatment scenarios [5]. Consequently, the identification of novel molecular biomarkers capable of more accurately predicting patient outcomes and informing therapeutic strategies is of paramount importance in advancing the management of this disease.

Actinin‐4, which is a member of the α‐actinin family, encoded by the *ACTN4* gene, is a crucial component of the cellular cytoskeleton, playing a fundamental role in the organization and dynamics of actin filaments [6]. By crosslinking actin filaments, actinin‐4 modulates a variety of cellular processes essential for cancer progression, including cell shape, adhesion, migration, and invasion [7]. Aberrant actinin‐4 expression has been implicated in the development and progression of various malignancies, including lung, breast, and colorectal cancers, where it has been associated with increased cell motilities and cancer invasion, often correlating with a less favorable patient prognosis [8, 9, 10, 11].

However, the precise role of actinin‐4 in endometrial carcinoma remains incompletely understood. To address this critical gap in knowledge, we conducted a comprehensive study to investigate the clinical significance of actinin‐4 expression in endometrial carcinoma. Utilizing immunohistochemical staining (IHC) and fluorescence in situ hybridization (FISH), we meticulously evaluated actinin‐4 protein expression and *ACTN4* gene amplification, respectively, within a large cohort of endometrial carcinoma patients. Subsequently, we rigorously correlated these findings with clinicopathological parameters and patient survival outcomes to determine the prognostic value of actinin‐4 in this malignancy.

## 2 Materials and Methods

### 2.1 Ethics Statement

The study protocol was approved by the Institutional Review Board of Nippon Medical School (IRB number: N‐2023‐099) [12] and complied with the ethical principles outlined in the Declaration of Helsinki.

### 2.2 Consent

Written informed consent was obtained from all patients prior to surgery.

### 2.3 Patients and Tissue Samples

This retrospective study included patients diagnosed with endometrial carcinoma who underwent surgical resection at Nippon Medical School Hospital between January 2018 and December 2022. Patients were included if they had histologically confirmed endometrial carcinoma and had not received any prior treatment, such as chemotherapy or radiotherapy, before surgery. Patients were excluded if they had received neoadjuvant therapy, had other synchronous malignancies, or if their tissue samples were inadequate for IHC and FISH analyses. Clinical and pathological data were collected, including age at diagnosis, International Federation of Gynecology and Obstetrics (FIGO) stage, histological subtype, tumor grade, depth of myometrial invasion (MMI), lymphovascular space invasion (LVSI), lymph node status, and available follow‐up information.

Patients were stratified into recurrence risk groups based on post‐operative pathological findings as follows: Low Risk included Stage IA endometrioid G1/G2 tumors with MMI < 1/2 and no LVSI. Intermediate Risk comprised Stage IA with LVSI, Stage IB endometrioid G1/G2, Stage IA endometrioid G3, and non‐endometrioid histological types (e.g., serous, clear cell, carcinosarcoma) without MMI. High Risk was defined as all non‐endometrioid histological types with MMI regardless of stage, Stage IB endometrioid G3, as well as any histology at Stage II or more [13].

### 2.4 Immunohistochemical Staining (IHC)

IHC was performed using a mouse monoclonal IgG anti‐actinin‐4 (Abnova, clone 13G9, dilution 1:300) as the primary antibody and a biotinylated horse anti‐mouse IgG antibody (H + L) (Vector Laboratories, Newark, CA) as the secondary antibody. Formalin‐fixed paraffin‐embedded tissue sections (4 μm thick) were stained using the immunoperoxide method as described previously [14]. Briefly, tissue sections were deparaffinized and subjected to antigen retrieval using heat citrate buffer (pH 6.0) at 121°C for 10 min. Endogenous peroxidase and biotin activities were blocked using 0.3% H_2_O_2_‐methanol and the Biotin‐Blocking System (Agilent Dako, Glostrup, Denmark, cat. #X0590). Subsequently, tissue sections were incubated with the primary antibody, diluted in 2% normal swine serum (NSS; Vector Laboratories), overnight at 4°C. The following day, sections were incubated with the secondary antibody for 1 h at room temperature. Tissue sections were then incubated with 3,3′‐diaminobenzidine (DAB) substrate and counterstained with Mayer's hematoxylin.

Stained sections were scanned using the Virtual Slide Scanner (NanoZoomer 2.0‐HT; Hamamatsu Photonics, Hamamatsu, Japan). Virtual tissue images were generated using the scanner software (NanoZoomer Digital Pathology Virtual Slide Viewer version 2.2; Hamamatsu Photonics, Hamamatsu, Japan) [14].

Actinin‐4 protein expression was assessed by two independent investigators (X.L. and Y.N.) blinded to the clinicopathological data. The intensity of actinin‐4 staining was graded as follows: Weak expression (Score 1+): tumor cells stained with weaker intensity than endothelial cells; Moderate expression (Score 2+): more than 30% of tumor cells stained with intensity equal to that of endothelial cells; or Strong expression (Score 3+): more than 30% of tumor cells stained with stronger intensity than endothelial cells.

### 2.5 Fluorescence In Situ Hybridization (FISH)

FISH was performed using a commercially available probe specific for the *ACTN4* gene (GSP Laboratory, Kawasaki, Japan) as described previously [9]. *ACTN4* gene copy number was assessed using an Axio Vision microscope (Carl Zeiss, Oberkochen, Germany). The numbers of fluorescence signals in at least 20 non‐overlapping nuclei per case were counted by two independent investigators (L.X. and Y.N.). FISH positivity was defined as an *ACTN4*/centromere probe for chromosome 19 (CEP19, GSP Laboratory) ratio of ≥ 2.0, and FISH negativity was defined as an *ACTN4*/*CEP19* ratio of < 2.0 [9].

### 2.6 Statistical Analysis

Statistical analysis was performed using SPSS version 29.0.2 (IBM Corp., Armonk, NY, USA) and R version 4.4.3. Associations between actinin‐4 status (evaluated by IHC and FISH) and clinicopathological features were assessed using appropriate statistical tests, including one‐way ANOVA for continuous variables. For survival analysis, actinin‐4 expression by IHC was categorized as low (Score 1+ or 2+) or high (Score 3+). This cut‐off was determined based on the Kaplan–Meier survival analysis, which showed that patients with an IHC score of 3+ had a significantly poorer prognosis, and thus most accurately reflected the prognostic significance of actinin‐4 expression. For *ACTN4* FISH analysis, cases were classified as having gene amplification or not. OS and PFS curves were estimated using the Kaplan–Meier method, and differences between groups were compared using the log‐rank test.

These analyses were conducted using the survival and survminer packages in R. Survival outcomes were further analyzed by comparing three groups based on the combined actinin‐4 IHC expression and FISH positivity of *ACTN4* status: (1) low IHC expression and no *ACTN4* gene amplification, (2) high IHC expression and no *ACTN4* gene amplification, and (3) high IHC expression and *ACTN4* gene amplification [15].

Univariate and multivariate analyses were performed using the Cox proportional hazards model to identify independent prognostic factors. A *p* value of < 0.05 was considered statistically significant.

## 3 Results

A total of 135 patients with endometrial carcinoma met the inclusion criteria for this study. The median age at diagnosis was 57 years, with a range from 28 to 83 years. The clinicopathological characteristics of the patient cohort are summarized in Table 1.

**TABLE 1:** Clinical and pathological characteristics of endometrial carcinoma patients.

Age at the start of treatment		57 (28–83)
FIGO stage (*N* = 135)	IA	88
	IB	22
	II	5
	III	13
	IV	7
Histology (*N* = 135)	G1	64
	G2	38
	G3	12
	Serous	12
	Clear	2
	Carcinosarcoma	4
	Dedifferentiated and undifferentiated	3
Chemotherapy regimen (*N* = 50)	TC	47
	DC	2
	AP	1
Number of administration cycles (*N* = 50)	6	44
	Other (1–8)	6
Recurrence risk group (*N* = 135)	Low	67
	Mid	27
	High	41

*Note:* G1/G2/G3, Endometrioid carcinoma Grade 1, Grade2, Grade3, respectively.

Abbreviations: AP: Doxorubicin and Cisplatin regimen; DC, Docetaxel and Carbopratin regimen; FIGO, International Federation of Gynecology and Obstetrics; TC, Paclitaxel and Carboplatin regimen.

**FIGURE 1:**
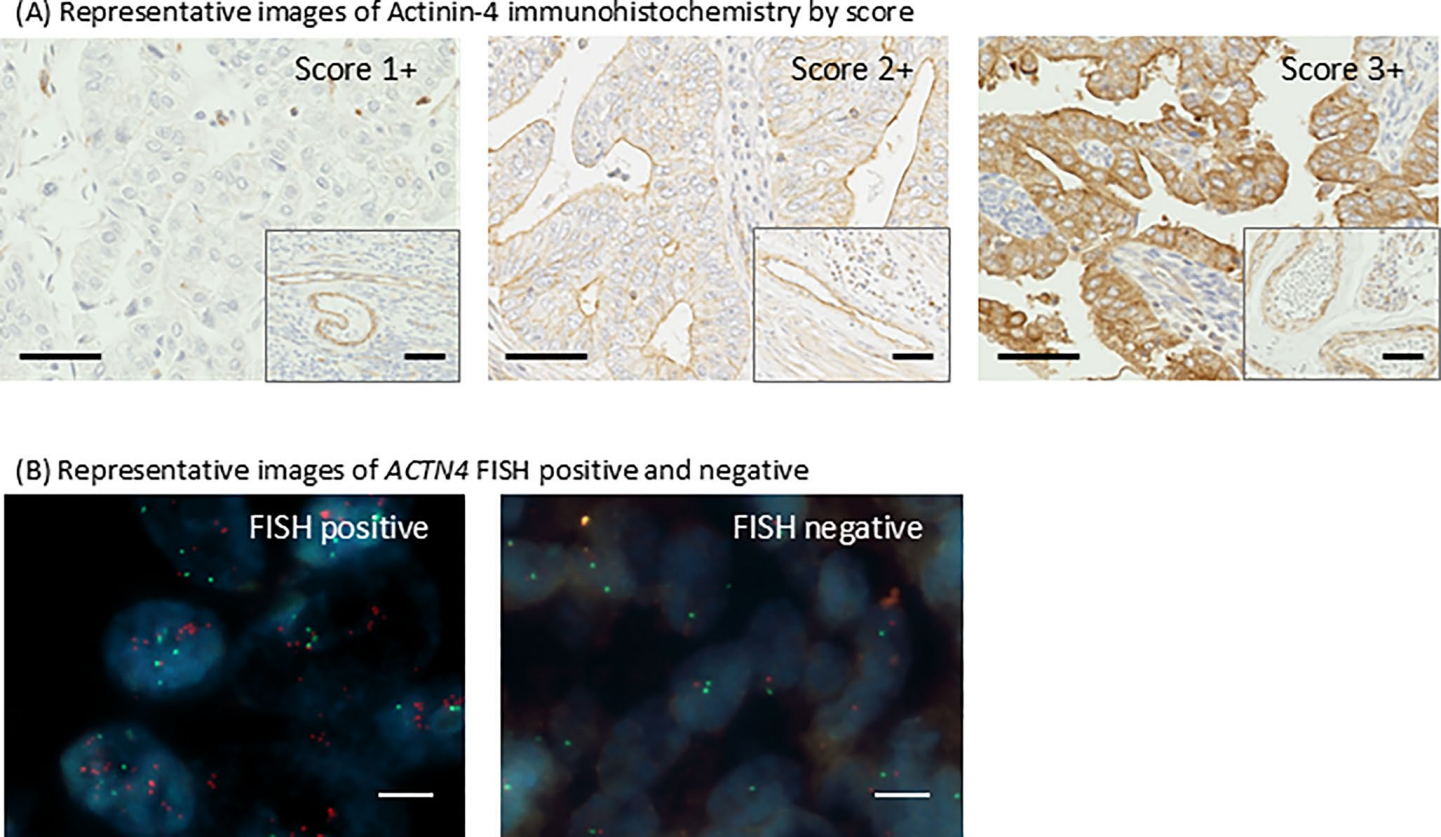
Representative images of actinin‐4 immunohistochemistry and *ACTN4* FISH. (A) Actinin‐4 staining was graded from score 1+ to 3+ based on the staining intensity within the tumor relative to that in vascular endothelial cells (within the lower right frame). Representative images of actinin‐4 immunohistochemistry by score are shown with scale bars. The black line scale in the figure is 50 μm. (B) Representative images of *ACTN4* FISH positive and negative are shown. The probe targeting *ACTN4* is labeled in red, and the chromosome 19 centromere probe is labeled in green. The white line scale in the figure is 5 μm.

Actinin‐4 protein expression was successfully evaluated by IHC in all 135 cases (Figure 1A). High actinin‐4 expression (Score 3+) was observed in 56 cases (41.5%), whereas low actinin‐4 expression (Scores 1+ or 2+) was observed in 79 cases (58.5%). A statistically significant association was found between high actinin‐4 protein expression and both advanced FIGO stage (*p* = 0.0065) and histological subtypes (*p* = 0.0041). No significant association was detected between actinin‐4 expression and recurrence risk group (*p* = 0.2984) (Table 2).

**TABLE 2:** The association between clinicopathological parameters and the expression levels of *ACTN4*.

		Actinin‐4 IHC score					*ACTN4* FISH +	Total
		1	2	3	Positive rate	*p*	Number	
FIGO stage (*N* = 135)	IA	11	45	32	36.40%	0.0065	4	88
	IB	2	10	10	45.50%		1	22
	II	2	1	2	40%		1	5
	III	0	6	7	53.80%		2	13
	IV	0	2	5	71.40%		1	7
Histology (*N* = 135)	G1	10	35	19	29.70%	0.0041	0	64
	G2	3	20	15	39.50%		0	38
	G3	1	5	6	50%		0	12
	Serous	0	2	10	83.30%		6	12
	Clear	1	0	1	50%		0	2
	Carcinosarcoma	0	0	4	100%		2	4
	Dedifferentiated and undifferentiated	0	2	1	33.30%		1	3
Recurrence risk group (*N* = 135)	Low	10	39	18	26.90%	0.2984	0	67
	Mid	3	12	12	44.40%		1	27
	High	2	13	26	63.40%		8	41

*Note:* G1/G2/G3, Endometrioid carcinoma Grade 1, Grade2, Grade3, respectively.

Abbreviation: FIGO, International Federation of Gynecology and Obstetrics.

*ACTN4* gene amplification was detected by FISH in 9 cases (6.7%) of the entire cohort (Figure 1B). While no amplification was found in any of the 102 G1/G2 endometrioid carcinoma cases, it was observed in 6 out of 12 cases of serous carcinoma and 2 out of 4 cases of carcinosarcoma (Table 2). Tumors exhibiting high actinin‐4 protein expression were more likely to show *ACTN4* amplification compared to tumors with low actinin‐4 expression; however, this trend did not reach statistical significance (*p* = 0.0629) (Table 3).

**TABLE 3:** Relationship between actinin‐4 IHC score and *ACTN4* FISH‐positive cases.

		*ACTN4* FISH +		Total
		Number	*p* value	
actinin‐4 IHC	Low (1+, 2+)	1	0.0629	79
	High (3+)	8		56

**FIGURE 2:**
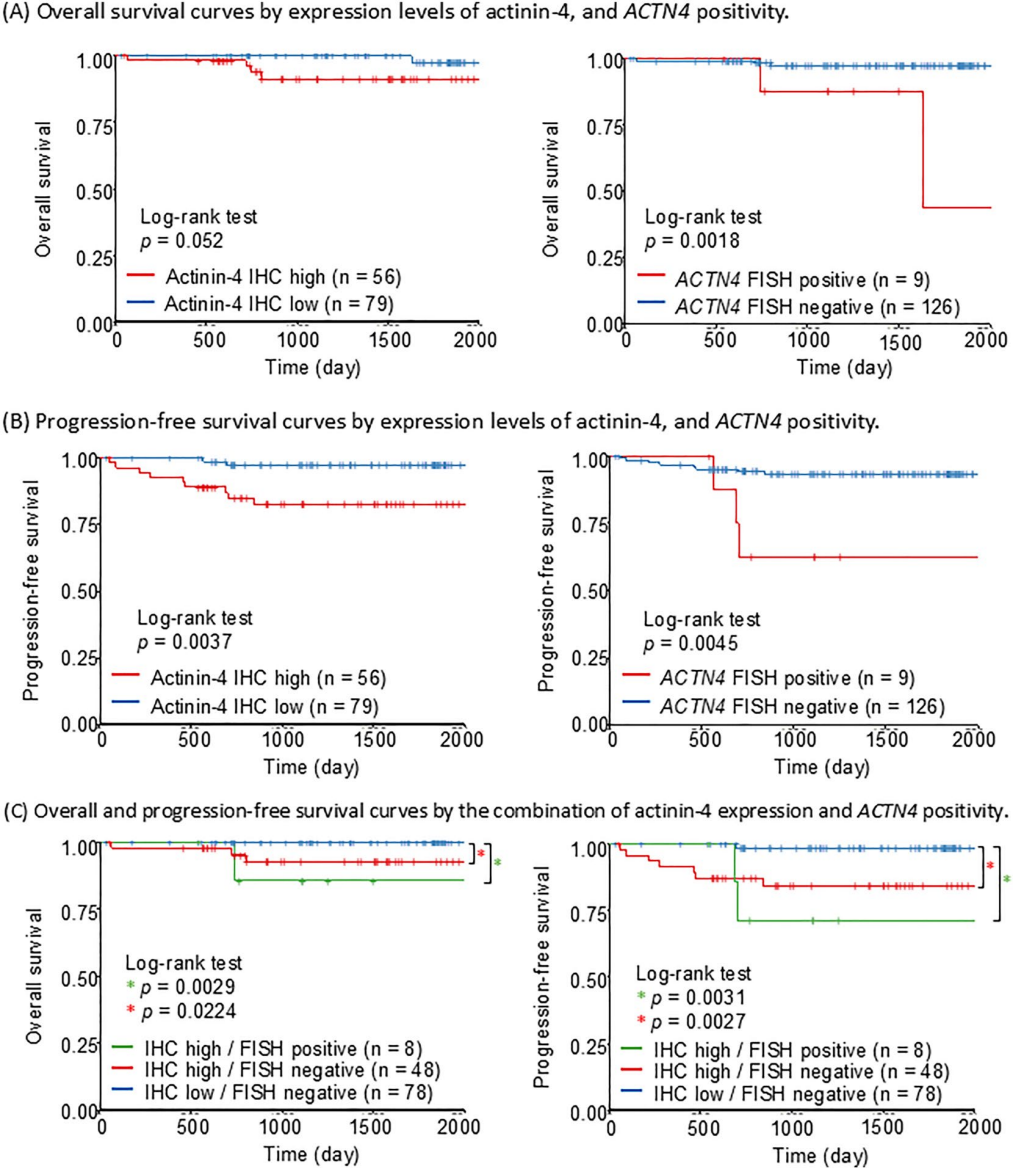
Kaplan–Meier curves for overall and progression‐free survival based on actinin‐4 expression and *ACTN4* amplification. (A) Overall survival curves illustrating outcomes based on actinin‐4 expression levels (left) and *ACTN4* amplification status (right). The case numbers (*n*) are provided for each group: High (*n* = 56) and low (*n* = 79) actinin‐4 IHC expression on the left, and *ACTN4* amplification (*n* = 9) and non‐amplification (*n* = 128) by FISH on the right. (B) Progression‐free survival curves illustrating outcomes based on actinin‐4 expression levels (left) and *ACTN4* amplification status (right). The case numbers (*n*) are provided for each group: High (*n* = 56) and low (*n* = 79) actinin‐4 IHC expression on the left, and *ACTN4* amplification (*n* = 9) and non‐amplification (*n* = 128) by FISH on the right. (C) Overall survival (left) and progression‐free survival (right) curves stratified by a combined assessment of actinin‐4 expression and *ACTN4* positivity. The case numbers (*n*) for each subgroup are detailed, including IHC high/FISH positive (*n* = 8), IHC high/FISH negative (*n* = 48), and IHC low/FISH negative (*n* = 78). *For actinin‐4 expression, low expression was defined as an IHC score of 1+ or 2+, and high expression as a score of 3+. For *ACTN4* gene amplification, cases were considered “positive” if the *ACTN4*/*CEP19* ratio was ≥ 2.0, and “negative” if the ratio was < 2.0.

In the Kaplan–Meier analysis of OS, no significant difference was observed between groups with high and low actinin‐4 expression by IHC (*p* = 0.052). In contrast, the group with *ACTN4* amplification by FISH showed a significantly worse prognosis (*p* = 0.0018) (Figure 2A). For PFS analysis, both high actinin‐4 expression by IHC (*p* = 0.0037) and *ACTN4* amplification by FISH (*p* = 0.0045) were significantly associated with poorer prognosis (Figure 2B). Furthermore, when comparing the three groups based on the combination of IHC and FISH findings, the group with low expression of actinin‐4 and no *ACTN4* amplification had a significantly better prognosis compared to the group with overexpression of actinin‐4 irrespective of *ACTN4* gene amplification status (Figure 2C).

In univariate analysis, *ACTN4* amplification by FISH and histological type were identified as poor prognostic factors for OS. However, no factors remained significant in multivariate analysis (Table S1). For PFS, univariate analysis revealed that high actinin‐4 expression by IHC, *ACTN4* amplification by FISH, FIGO stage, and histological type were poor prognostic factors. In multivariate analysis, FIGO stage was the only remaining independent poor prognostic factor (Table S2).

## 4 Discussion

This study investigated the clinical significance of actinin‐4 expression in endometrial carcinoma by evaluating both protein expression via IHC and gene amplification using FISH. Our findings provide several key insights into the role of actinin‐4 in this malignancy.

Firstly, we observed a significant association between high actinin‐4 protein expression and aggressive clinicopathological features, specifically advanced FIGO stage (*p* = 0.0065) and histological subtypes (*p* = 0.0041). This aligns with previous studies in other cancer types where actinin‐4 overexpression has been linked to increased tumor aggressiveness and metastatic potential [8, 9, 10]. The correlation between actinin‐4 overexpression and aggressive tumor features may be attributed to actinin‐4's role in cytoskeletal dynamics, where it contributes to cell motility and invasion, key processes in cancer progression [16]. This enhanced motility may facilitate the spread of tumor cells through the myometrium and lymphatic system, potentially leading to lymph node metastasis and advanced disease stage [17].

Based on our univariate analysis, *ACTN4* gene amplification was a significant prognostic factor for both OS and PFS. However, it was not found to be an independent prognostic factor in the multivariate analysis. While the established prognostic factor of advanced FIGO stage was an independent prognostic factor for PFS in the multivariate analysis, we could not perform the same analysis for OS due to a limited number of events. Given the event frequency in our cohort, a larger study, such as a multi‐institutional trial, is required to definitively determine if high *ACTN4* expression or gene amplification is an independent prognostic factor.

The observation that *ACTN4* amplification appears to be more frequent in serous carcinoma and carcinosarcoma, which are histological subtypes often associated with the copy‐number high (CN‐H) molecular subtype in TCGA classification. The overlap of *ACTN4* amplification with aggressive histological subtypes makes it difficult to definitively determine whether *ACTN4* amplification is an independent driver of tumor aggressiveness or a surrogate marker of this phenotype.

Our findings align with previous research in ovarian cancer, specifically serous adenocarcinoma, where *ACTN4* gene amplification has been linked to increased actinin‐4 protein immunoreactivity [18]. This consistency reinforces the potential role of *ACTN4* amplification and subsequent actinin‐4 expression in driving the aggressive characteristics of this tumor subtype. While we conducted a subgroup survival analysis on the serous carcinoma and carcinosarcoma cases, the limited number of patients precluded any definitive conclusions. Therefore, a larger cohort study is necessary to determine if *ACTN4* amplification provides additional prognostic value beyond histology alone in these aggressive subtypes.

Secondly, analysis using Kaplan–Meier curves revealed differential prognostic significance for actinin‐4 based on the assessment method. Overexpression of actinin‐4 protein that was evaluated by IHC was significantly associated with a poorer prognosis in terms of PFS (*p* = 0.0037), but this association did not reach the conventional threshold for statistical significance regarding OS (*p* = 0.052). In contrast, *ACTN4* gene amplification, as identified by FISH, demonstrated a statistically significant association with a poorer prognosis for both OS (*p* = 0.018) and PFS (*p* = 0.0045). The observed discrepancy in the significance of OS between the IHC and FISH analyses, particularly the borderline result for IHC OS, can be attributed to the limited number of death events in our uterine cancer cohort.

Our analysis of *ACTN4* gene amplification using FISH presented a complex picture. Although we observed a higher frequency of *ACTN4* gene amplification in tumors with high protein expression, the correlation was not absolute. This finding suggests that, in addition to gene amplification, other non‐amplification‐related mechanisms are crucial for driving high actinin‐4 protein expression in a significant subset of endometrial carcinoma patients. We hypothesize that these mechanisms include a variety of regulatory processes at the transcriptional, post‐transcriptional, and post‐translational levels. At the transcriptional level, increased gene expression could be driven by specific transcription factors.

Additionally, epigenetic regulation, including DNA hypomethylation in the *ACTN4* promoter region or specific histone modifications, could lead to a more open chromatin state and enhanced transcription. Post‐transcriptionally, the stability of *ACTN4* mRNA could be a key factor. MicroRNAs (miRNAs) are known regulators of gene expression, and certain miRNAs might be downregulated in these tumors, leading to prolonged *ACTN4* mRNA half‐life and subsequent protein overexpression. At the post‐translational level, the degradation rate of the actinin‐4 protein could be altered. Impaired ubiquitination and proteasomal degradation pathways could result in the accumulation of actinin‐4 protein, independent of its synthesis rate. Future research is warranted to elucidate these intricate regulatory mechanisms.

Our study has certain limitations. Firstly, the retrospective nature of the study may introduce inherent biases. Data on p53 IHC or surrogate molecular classification was not available, which limited our ability to comprehensively evaluate the relationship between *ACTN4* amplification and the molecular characteristics of the tumors. Future studies should focus on integrating these molecular classifications to further clarify the role of *ACTN4* as a prognostic marker. Furthermore, treatment decisions were not standardized as in clinical trials and varied according to individual physicians' practices, which could have confounded the association between actinin‐4 expression and survival outcomes.

Secondly, the lack of standardized scoring systems for actinin‐4 IHC could lead to some degree of interobserver variability. Although we employed two independent pathologists to review and score the IHC results and reached a consensus in cases of discrepancy, the semi‐quantitative nature of IHC scoring inherently involves subjective interpretation, which can affect the reproducibility of the findings across different laboratories. The IHC scoring criteria for actinin‐4 expression in the present study differ from those utilized in our previous works, in which both scores 2+ and 3+ were classified as actinin‐4 positive due to the limited number of cases exhibiting low actinin‐4 expression [9]. Nevertheless, based on our Kaplan–Meier survival analysis, classifying a score of 3+ as positive is the optimal cut‐off for predicting PFS, which provides a robust rationale for our chosen scoring method. These findings suggest that the IHC pattern for actinin‐4 in endometrial carcinoma may differ from that in other tumor types.

Thirdly, the relatively limited sample size in some of the subgroups may have reduced our statistical power to detect subtle but potentially relevant associations. Specifically, the number of patients with certain histological subtypes or advanced FIGO stages might have been insufficient to demonstrate statistically significant differences in survival, even if a clinically meaningful trend existed.

Despite these limitations, our study offers valuable insights into the clinical significance of actinin‐4 in endometrial carcinoma. The combined use of IHC and FISH analyses allowed us to demonstrate that high actinin‐4 protein expression is associated with more aggressive tumor behavior, particularly concerning PFS. Moreover, exploring the potential of actinin‐4 as a therapeutic target represents a promising avenue for future research, especially for patients exhibiting high actinin‐4 expression and aggressive disease characteristics.

In conclusion, our findings suggest that actinin‐4 plays a significant role in the progression of endometrial carcinoma, influencing tumor aggressiveness and potentially patient outcomes. The significant association between *ACTN4* amplification and poor prognosis suggests its potential clinical utility. However, it is crucial to validate actinin‐4's prognostic value in larger, independent prospective cohorts to confirm its clinical utility. Future research should explore the therapeutic potential of targeting actinin‐4 in endometrial carcinoma. This could involve in vitro studies using endometrial cancer cell lines to investigate the effects of *ACTN4* knockdown or pharmacological inhibition on key cancer‐related processes such as cell proliferation, migration, and invasion. In vivo studies using xenograft or genetically engineered mouse models would then be essential to assess the efficacy and safety of actinin‐4‐targeted therapies. Ultimately, these preclinical investigations may pave the way for clinical trials to evaluate their safety and efficacy in patients, leading to more personalized and effective management strategies for endometrial carcinoma.

## Author Contributions

**Li Xiang:** data curation, investigation. **Yutaka Naito:** project administration, data curation, conceptualization, methodology, investigation, validation, visualization, writing – original draft, writing – review and editing. **Masafumi Toyoshima:** conceptualization, methodology, data curation, investigation, validation, visualization, writing – original draft, writing – review and editing, project administration. **Mika Terasaki:** data curation, formal analysis, resources. **Akihito Yamamoto:** data curation. **Akira Shimizu:** data curation, resources, formal analysis. **Shunji Suzuki:** supervision. **Kazufumi Honda:** writing – review and editing, supervision, funding acquisition, resources, conceptualization.

## Disclosure

The authors have nothing to report.

## Conflicts of Interest

The authors declare no conflicts of interest.

## Supporting information


**Table S1:** Univariate and Multivariate Analysis of Factors Associated with Overall Survival using the Cox proportional hazards model.

**Table S2:** Univariate and Multivariate Analysis of Factors Associated with Progression Free Survival using the Cox proportional hazards model.

## Data Availability

The data that supports the findings of this study are available in Supporting Information of this article.
